# Plasmatic markers for early diagnostic and treatment decisions in ischemic stroke

**Published:** 2015

**Authors:** DM Stanca, IC Mărginean, O Sorițău, DF Mureșanu

**Affiliations:** * Department of Neurology, University of Medicine and Pharmacy Cluj-Napoca, Romania; ** Department of Cancer Immunology, “Prof. Dr. Ion Chiricuta” Cancer Center, Cluj-Napoca, Romania

**Keywords:** neuronal biomarker, ischemic stroke, early diagnosis, biomarker panel

## Abstract

A neurologic deficit of sudden onset conforming to a vascular territory is a clear clinical indication that a patient suffers from an acute stroke. However, the imagistic diagnostic confirmation is not always readily available. We are now able to offer comprehensive medical support for the patient after an acute stroke and to make a prodigious rehabilitation program after the damage is done, but this is not offering the chance for improvement. An opportunity to better diagnose ischemic stroke seems to be available by using neuronal biomarkers. Extensive research is being conducted in this field and useful information is beginning to gather. This mini-review aims to highlight selected studies that appear to be of particular interest for the clinical neurologist. The most promising biomarkers (or rather panels of biomarkers) are presented with theirs clinical usefulness and limitations.

A neurologic deficit of sudden onset conforming to a vascular territory is a clear clinical indication that a patient is suffering from an acute stroke. For such a patient it is in theory a rather simple procedure to have a confirmation of the diagnostic in a hospital setting, by using cerebral imaging. An intracerebral hemorrhage is readily discernable with cerebral imaging; however, it is a different thing with cerebral ischemia. The therapeutic window leaves precious little time when the diagnosis of acute cerebral ischemia is established with certainty.

Neuro-imaging is considered to date the only tool available for differentiating between ischemic stroke and intracerebral hemorrhage, as the symptoms of the two conditions show substantial overlap. We have two options when a patient in whom we suspect an acute ischemic stroke is brought in the Emergency Department: either we give thrombolytic therapy (provided we are within 4 and a half hours after the onset of symptoms and we have strong indicators that the patient is having an ischemic event) or we offer supportive treatment. Since ischemia is visible with cerebral CT in the first 3 hours after the onset in only a third of the cases [**1**], there is a clear need for other diagnostic tools and strategies to make the differential diagnosis between ischemia and hemorrhage. Such an opportunity seems to be offered by the neuronal biomarkers [**2**].

They are substances found in the neural tissue and are rapidly released into the blood stream after neuronal injury. If their blood levels should correlate with the type and extent of the neural damage, we would be in the position of supplementing our diagnostic capabilities with tests that could be performed in the pre-hospital setting or the outpatient unit, thus putting us in the position of offering the thrombolytic therapy as early as possible [**3**].

Brain ischemia induces cellular energetic failure, excitotoxicity, oxidative stress, erratic enzymatic activation, microvascular injury and cell death, events that constitute the ischemic cascade [**4**]. The permeability of the blood-brain barrier during the first 48 hours after onset is influenced by the extracellular enzymatic activation; further damage might occur during reperfusion [**5**].

In case of an intracerebral hemorrhage, the neuronal injury is supplemented by the compressive effect of the hematoma, the systemic inflammatory response, the neuronal toxicity of the hemoglobin and the activation of the thrombolysis within the cerebral clot. The fact that the brain tissue damage is caused through different mechanisms in the two types of stroke may offer us the chance of discriminating between the two of them by using circulating neuronal biomarkers [**6**,**7**].

For decades, researchers have studied dozens of blood proteins in their role of neuronal biomarkers and have published their results. A limited number of substances are constantly cited as having significance for the diagnostic of ischemic stroke.

**Matrix metalloproteinases (MMPs)** are a family of zinc- and calcium-dependent endopeptidases responsible for turnover and degradation of extracellular matrix proteins. Cerebral tissue expression of MMP-9 is normally minimal, but increases in MMP-9 were discovered in the ischemic brain [**8**,**9**]. They have a dual role during ischemic stroke: a deleterious one in the acute phase, when they are responsible for the disruption of the blood–brain barrier, neuronal cell death and hemorrhage after stroke, and a healing role during brain regeneration and neurovascular remodeling in the later tissue repair phase. Although the blood level of the MMPs rises in the wake of both ischemic and hemorrhagic stroke, the MMP-9 blood concentrations are best characterized in ischemic lesions. They have been identified as a predictor of infarct volume as measured with diffusion-weighted MRI [**10**] and the biomarker is further correlated with the effective application of thrombolytic therapy [**11**]. Blood MMP-9 concentrations in stroke patients treated with rtPA were significantly higher than those in untreated patients [**12**]. Hyper acute MMP-9 blood concentrations are considered a predictor of further hemorrhagic complications after rtPA administration.

**Apolipoprotein CIII** (Apo CIII) is present in normal plasma at 0.1 g/ L and is mainly found in VLDL but also in HDL and LDL particles. It is associated with atherogenesis both by lipid mechanisms, direct effects on endothelial cells (by stimulating the adhesion of monocytes to the endothelial bed), and endothelial dysfunction [**13**]. Apo CIII is currently considered an important factor in the atherosclerotic phase of the cardiovascular disease; its role in late, more acute, thrombotic events or as a neuronal biomarker in stroke has been less explored so far [**14**]. 

**S100B** is a low molecular weight glial protein that belongs to a multigenic family of calcium-mediated proteins and has its name derived from the fact that the protein is 100% soluble in ammonium sulfate at neutral pH. Several studies have demonstrated that serum S100B concentrations are increased significantly following stroke [**15**,**16**].

It seems though that this marker should better be used for its prognostic value following stroke rather than for acute differential diagnostic. After transient ischemic attacks, the blood levels of S100B have minimal variations, while during stroke the serum levels rise, with a peak after 24 hours [**17**]. Furthermore, the blood levels of S100B correlate with the extent of the brain lesion and with outcome. The peak and the area under curve levels correlated in a recently published study with subacute infarct volume and the correlations were stronger when measured after 24 h than closer to admission [**18**]. 

The substance was also studied as a marker for brain damage during surgical interventions (as a biochemical marker of brain ischemic damage after the treatment of carotid stenosis). In a series of patients treated for atherosclerotic carotid stenosis there were 16 patients with increased postoperative levels of S100B: three affected by postoperative stroke, two patients with minor stroke, one patient with fatal stroke and 12 patients with uneventful neurological outcome and positive brain imaging. The blood levels of S100B returned to normal within the first 24 hours for the patients with positive brain imaging and without clinical manifestations, after 120 and 144 hours for the patients with minor stroke and it never returned to normal for the patient with the fatal stroke [**19**]. 

In the first few hours after the onset of stroke, high blood levels of S100B should be seen as a marker of brain-blood barrier dysfunction rather than as an indicator of infarct size, as the latter is not yet established at this early point in time. Therefore, S100B may be used as a predictor for the hemorrhagic transformation of an ischemic stroke [**20**]. Furthermore, studies have shown that S100B was significantly increased after stroke onset compared with controls and correlated with the infarct volume, stroke severity and functional outcome. High blood levels that fail to normalize after more than 120 hours are regarded as a possible marker for ongoing ischemia [**21**]. 

**The Neuron-Specific Enolase (NSE)**, the isoenzyme yy of the enolase is a phosphopyruvate hydratase, a metalloenzyme responsible for the catalysis of the conversion of 2-phosphoglycerate to phosphoenolpyruvate, the ninth and penultimate step of glycolysis. It has been studied for decades as a biomarker for cerebral tissue damage [**22**] and most studies showed that the peak levels of NSE in serum were found within the first 96 hours after cerebral infarction [**23**,**24**]. Peak level of NSE best reflects the final infarct volume and thus carries mainly a prognostic value, its use during the first hours after onset being unrewarding. Serial measurements of NSE levels seem to be useful markers for ongoing brain ischemia [**25**]. 

**N-methyl-D-aspartic acid (NMDA) receptor** binds the glutamate neurotransmitter; after neuronal death fragments of this receptor and later antibodies against them are released into the blood stream. Whereas the synthesis of the antibodies requires a certain amount of time and thus limiting the use during the acute phase, NR1 and NR2 subunits of the receptor are readily released into the blood stream after the onset of stroke. The fragmentation of the NR2 subunit into the peptides NR2A and NR2B is caused by ischemia or neurotoxicity, thus offering the chance to discriminate between ischemic and hemorrhagic events [**26**]. The NMDA receptor peptide is an indicator of glutamate excitotoxicity associated with acute stroke patophysiology and can be used in the diagnosis of acute ischemic stroke within 3 hours from symptom onset.

**The combination of clinical tests and blood biomarkers** was also extensively studied. The markers t-PA and NT-proBNP were associated in a recent study positively and significantly (p < 0.01) with a diagnosis of transient cerebral ischemia or stroke and a model containing the FAST (Face Arm Speech Test), age, systolic blood pressure, NT-proBNP and t-PA had a better sensitivity (88%, p < 0.006) and a better specificity (48%, p = 0.04) than the FAST test alone. The authors concluded that the panels of biomarkers may marginally improve diagnosis, but their practicability is uncertain, and requires further study [**27**]. 

The proteins released from cerebral ischemic tissue were studied in an experimental design in an excellent study recently published [**28**]. More than 2200 proteins were identified from the ischemic hemisphere with < 1.0% false discovery rate; the data were processed with data mining technologies and proteins having an involvement in the energy metabolism (Pygb, Atp5b), glutamate excitotoxicity (Slc1a3, Glud1), neuro-inflammation (Tf, C3, Alb), and cerebral plasticity (Gfap, Vim, Gap43) were cited as having a diagnostic value for ischemic stroke. Several regulated proteins (Caskin1, Shank3, Kpnb1, Uchl1, Mtap6, Epb4.1l1, Apba1, and Ube1x) novel in the context of stroke were also discovered.

Clinical-diffusion mismatch (CDM; National Institutes of Health Stroke Scale score ≥ 8 and diffusion-weighted imaging lesion volume < 25 mL) has been suggested as a surrogate of ischemic brain at risk of infarction and might be used to recognize salvageable ischemic tissue. High levels of interleukin-10, tumor necrosis factor-α, and glutamate as well as low levels of neuron-specific enolase, interleukin-6, and active matrix metalloproteinase-9 are associated with CDM, thus offering diagnostic and prognostic information [**29**]. 

Blood measurements of biomarkers of brain damage and activation of the coagulation system may potentially serve as novel diagnostic tools for stroke subtypes. Ninety-seven stroke patients were prospectively investigated and the combination of glial fibrillary acidic protein (GFAP) and it was discovered that activated protein C-protein C inhibitor complex (APC-PCI) levels in patients with NIHSS score of more than 3 had a sensitivity and negative predictive value of 100% for ruling out intracerebral hemorrhage [**30**].

In a pilot study for the diagnosis of ischemic stroke within 6 hours from symptom onset that included brain natriuretic peptide, C-reactive protein, D-dimer, MMP-9, and protein S100B, a sensitivity of 81% and a specificity of 70% in diagnosing ischemic stroke, were recorded [**31**]. However, these encouraging results were not validated in prospective multicentre trials. After assessing the data from more than 1,100 patients presenting with symptoms suspicious for stroke with multivariate analysis, the authors concluded that the panel was capable of only moderately differentiating between stroke patients (IS, ICH) and mimics, with a sensitivity of 86% and a specificity of 37% for the discrimination of stroke patients from mimics. The diagnostic accuracy of this biomarker panel was clearly imperfect. Nevertheless, they claimed that a point-of-care algorithm might be feasible to aid in the early management of patients with symptoms suspicious for stroke [**32**].

Plasma samples from 223 stroke patients (including ischemic stroke, intracerebral hemorrhage and subarachnoid hemorrhage) and from 214 healthy individuals were screened for more than 50 serum biomarkers. Astroglial protein S100B, B-type neurotrophic growth factor, von Willebrand factor (vWF), matrix metalloproteinase-9 (MMP-9), and monocyte chemotactic protein-1 were identified to be associated with stroke. The data on 65 patients with suspected ischemic stroke and 157 healthy controls revealed that protein S100B, MMP-9, vascular cell adhesion molecule and vWF were identified as being associated with ischemic stroke. The combined model revealed both a 90% sensitivity and specificity for predicting ischemic stroke [**33**,**34**]. 

The field of stroke biomarkers is about to take a huge leap due to advances in proteomics and genomics [**35**]. The new fields of research are the peripheral blood mononuclear immune cells, messenger RNA expression studies and oligonucleotide microarrays. Multiple gene expression studies of the activation of the immune system show promise in identifying markers of the ischemic cascade activated by acute stroke. Peripheral blood lymphocytes are key players in the evolution of brain tissue injury in ischemic stroke and are selectively activated to infiltrate the ischemic brain areas. These inflammatory immune cells exacerbate ischemic reperfusion and also mediate cell repair and tissue remodeling [**36**]. 

Messenger RNA expression studies have recently been introduced in the field of acute stroke. The potential for discovery of new candidate biomarkers is highly promising. In a mouse model, virsinin-like protein 1 was identified as a potential candidate for an acute blood-borne marker of ischemic stroke [**37**]. PARK7 protein and nucleoside diphosphate kinase A in the spinal fluid were identified as candidates for ischemic stroke biomarkers. Ischemic stroke could be diagnosed by PARK7 with a sensitivity of 58% and specificity of 90% and by nucleoside diphosphate kinase A with a sensitivity of 67% and a specificity of 90% [**38**].

Reproducible gene expression patterns within 24 hours after ischemic stroke have been identified [**39**]. The genes expressing amphiphysin and interleukin-1 receptor 2 are up-regulated after intracranial hemorrhage and can help identifying ischemic stroke subtypes. Lymphocyte gene expression along with circulating stem cells is involved in tissue repair and recovery after stroke [**40**].

Hemorrhagic transformation is a significant complication following ischemic stroke. The identification of patients at increased risk of hemorrhage could help reduce the incidence of this complication and potentially allow the extension of the time window for t-PA administration in selected patients [**41**]. Several biomarkers have also been associated with an increased risk of hemorrhage following administration of tPA, including MMP-9, c-FN, PAI-1, TAFI and S100B.

Cellular fibronectin (c-Fn) is another factor that has been associated with increased hemorrhagic transformation. Cellular-Fn is synthesized by endothelial cells and is elevated following vascular injury including ischemic stroke. A study showed c-Fn levels > 3.6ug/ ml predict the development of hemorrhagic transformation following tPA use with a sensitivity of 100% and specificity of 96% [**42**]. 

## Conclusions

Biomarkers can play many useful roles in neurology. Early diagnosis and immediate therapeutic interventions are important factors to reduce the extent of brain tissue damage in case of ischemic stroke and the risk of stroke-related death. The clinical relevance of all candidate markers needs to be carefully validated in future controlled clinical trials with analysis geared toward focused clinical questions. The field of neuronal biomarkers currently offers a chance of improving the results of the medical management of acute ischemic stroke.

Stroke biomarkers await prospective validation studies. Clinical utility and cost-effectiveness need to be established before biomarkers can be used routinely in clinical practice. The neurologists wait for evidence on whether the use of the neuronal biomarkers can improve patient care.

**Acknowledgments**

This work was supported by CNCSIS –UEFISCSU, project number PNII – IDEI 2621/ 2008.
